# Association between gestational weight gain and adverse pregnancy outcomes in advanced maternal age: a retrospective cohort study

**DOI:** 10.1186/s12884-026-09596-y

**Published:** 2026-07-20

**Authors:** Shan Zhao, Sun Guo, Chenhong Cao, Lele Wang, Furong Xing, Jianya Ye, Xue Cong, Chongkai Wang

**Affiliations:** 1 Faculty of Nursing, Hebei University of Chinese Medicine, Shijiazhuang, China; 2 Hebei Provincial Hospital of Traditional Chinese Medicine, Shijiazhuang, China; 3 Faculty of Nursing, Tianjin University of Traditional Chinese Medicine, Tianjin, China; 4 The Chinese People’s Armed Police Force Hebei Provincial Corps Hospital, Shijiazhuang, China

**Keywords:** Advanced maternal age, Gestational weight gain, Pregnancy outcome, Low birth weight, Macrosomia

## Abstract

**Objective:**

To investigate the association between gestational-age-adjusted gestational weight gain (GWG) and adverse maternal and neonatal outcomes among women of advanced maternal age (AMA).

**Methods:**

This retrospective cohort study included 714 singleton pregnant women aged 35 years or older who received antenatal care and delivered at Hebei Provincial Hospital of Traditional Chinese Medicine between January 2022 and December 2025. GWG was calculated as the difference between pre-delivery weight and pre-pregnancy weight. Based on the Chinese standard WS/T 801–2022, participants were classified into insufficient, adequate, and excessive GWG groups using gestational-age-adjusted recommended ranges according to pre-pregnancy body mass index. Baseline characteristics, pregnancy complications, maternal outcomes, and neonatal outcomes were compared across GWG categories. Multivariable logistic regression models were used to estimate the associations between GWG categories and adverse outcomes, with adequate GWG as the reference group.

**Results:**

Among the 714 women included in the analysis, 112 (15.7%) had insufficient GWG, 282 (39.5%) had adequate GWG, and 320 (44.8%) had excessive GWG. After adjustment for potential confounders, insufficient GWG was associated with increased risks of premature rupture of membranes (aOR = 1.65, 95% CI: 1.01–2.69), gestational hypothyroidism (aOR = 1.89, 95% CI: 1.08–3.30), and low birth weight (aOR = 2.12, 95% CI: 1.05–4.29). Excessive GWG was associated with increased risks of cesarean delivery (aOR = 1.47, 95% CI: 1.04–2.08), postpartum anemia (aOR = 1.78, 95% CI: 1.12–2.84), preeclampsia (aOR = 1.71, 95% CI: 1.01–2.89), and macrosomia (aOR = 2.12, 95% CI: 1.16–3.88). The association between GWG category and preterm birth was attenuated after adjustment. Sensitivity analysis restricted to term pregnancies yielded generally consistent results.

**Conclusion:**

Among women of advanced maternal age, both insufficient and excessive gestational-age-adjusted GWG were associated with increased risks of adverse maternal and neonatal outcomes. These findings suggest that individualized monitoring and management of GWG during pregnancy may help improve pregnancy outcomes in this high-risk population.

## Take-home message

Gestational weight gain outside the recommended range is common among women of advanced maternal age and is associated with an increased risk of adverse pregnancy outcomes.Optimizing weight management during pregnancy may be an important strategy to improve maternal and neonatal outcomes in this high-risk population.

## Introduction

Advanced maternal age (AMA), commonly defined as maternal age ≥ 35 years at delivery, has become increasingly prevalent worldwide [1, 2]. In recent decades, socioeconomic development, changes in reproductive attitudes, and advances in assisted reproductive technologies have contributed to a continuous postponement of childbearing among women. Epidemiological studies have shown that the average maternal age has increased substantially in many high-income countries over the past decades [3]. For instance, in Spain, the mean maternal age at childbirth increased from 26 years in 1978 to 32 years in 2017 [4], and similar demographic trends have been observed in other European countries. A comparable pattern has also emerged in China. In China, a comparable trend has also been observed. Following the implementation of the universal two-child and three-child policies, the proportion of women of advanced maternal age increased from approximately 12.5% during the one-child policy period to more than 21% in recent years [5]. 

In addition, population-based studies have reported a gradual increase in maternal age in several regions of China. For example [6], a registry-based study from central China showed that the mean maternal age increased from 27.1 to 29.7 years, while the proportion of pregnant women aged ≥ 35 years rose from 4.3% to 13.9%. These demographic changes suggest that AMA has become an increasingly important factor influencing maternal and perinatal outcomes.

Pregnant women of AMA are more likely to have pre-existing chronic conditions, such as hypertension and diabetes, which increase the risk of pregnancy complications. Previous studies have shown that the incidence of hypertensive disorders of pregnancy and gestational diabetes mellitus is significantly higher in women with advanced maternal age than in younger women [7]. Furthermore, advanced maternal age has been associated with an increased risk of preterm birth, cesarean delivery, and other adverse perinatal outcomes [8], making pregnancy management in this population more complex and challenging.

Because AMA is associated with a higher baseline risk of pregnancy complications, identifying modifiable risk factors in this population is clinically important.Among the numerous factors influencing pregnancy outcomes, gestational weight gain (GWG) is an important and modifiable one [9]. Excessive GWG has been associated with an increased risk of macrosomia, cesarean delivery, and gestational hypertension, as well as postpartum weight retention and long-term metabolic disturbances. Conversely, insufficient GWG is closely linked to fetal growth restriction, low birth weight, and preterm birth. Abnormal GWG may also affect neonatal short-term health and long-term development, including an elevated risk of obesity, insulin resistance, and cardiometabolic disorders [10–12]. Weight management may be particularly challenging in women of advanced maternal age because age-related metabolic changes, hormonal alterations, and a higher burden of chronic diseases may contribute to abnormal GWG and adverse pregnancy outcomes.

Although previous studies have investigated the relationship between GWG and pregnancy outcomes, evidence focusing specifically on focusing on women of AMA, particularly within the Chinese population, remains limited. Most existing research has primarily concentrated on the general pregnant population, and the specific patterns of GWG and their impact on maternal and neonatal outcomes among older women have not been fully characterized. Against this background, the present study retrospectively analyzed GWG patterns in women of AMA and further examined the associations between abnormal weight gain and pregnancy outcomes. The findings aim to provide evidence-based guidance for weight monitoring and management in this high-risk population, as well as to inform clinical intervention strategies, ultimately contributing to the reduction of adverse maternal and neonatal outcomes.

## Method

### Study population

This retrospective study included pregnant women aged ≥ 35 years at delivery who received antenatal care and delivered at Hebei Provincial Hospital of Traditional Chinese Medicine between January 2022 and December 2025. Eligible participants were required to have a singleton pregnancy, at least four antenatal visits at the hospital, and available data on pre-pregnancy weight and pre-delivery weight for GWG calculation. Women were excluded if they had severe pre-existing chronic diseases before pregnancy, fetal congenital anomalies, iatrogenic preterm birth due to severe maternal complications, or incomplete clinical data. Iatrogenic preterm birth was defined as preterm delivery initiated for medical indications, such as severe preeclampsia, severe placental abruption, or other life-threatening maternal or fetal conditions.

**Fig. 1:**
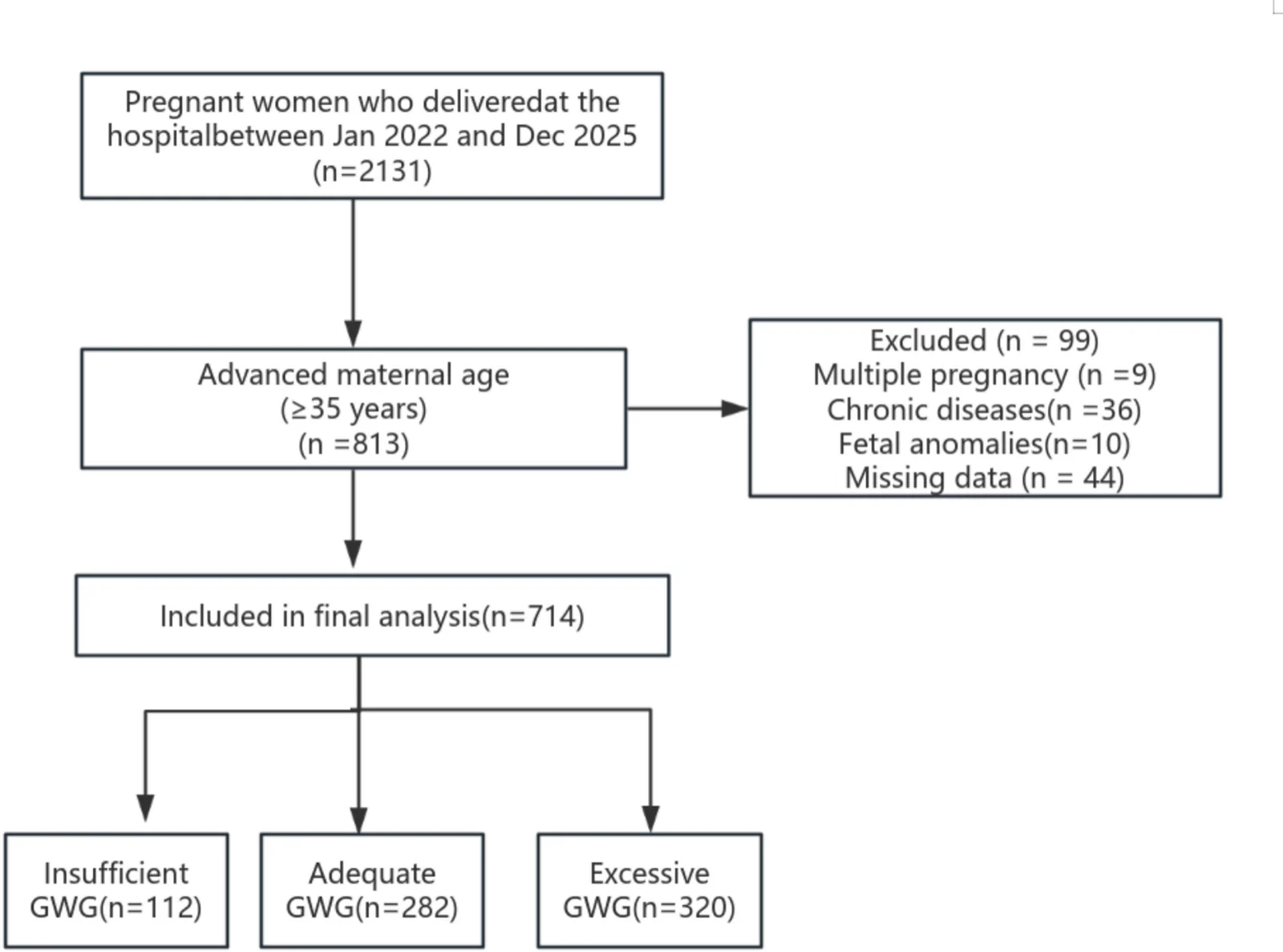
Flow chart of the study design

Finally, 714 eligible women were included in the analysis (Fig. 1). The study was approved by the Ethics Committee of Hebei Provincial Hospital of Traditional Chinese Medicine (Approval No. HBZY2026-KYLL-005-01). As this was a retrospective study based on routinely collected clinical data, the requirement for individual informed consent was waived by the same Ethics Committee.

### Definition and classification of gestational weight gain

GWG was defined as weight before delivery minus pre-pregnancy weight and was expressed in kilograms. According to WS/T 801–2022 [13], pre-pregnancy weight was defined as the average weight within 3 months before pregnancy, and weight before delivery was defined as the last weight measured within 1 week before delivery. Pre-pregnancy BMI was calculated as pre-pregnancy weight in kilograms divided by the square of height in meters.

Participants were categorized according to Chinese adult BMI cutoffs as follows: underweight, BMI < 18.5 kg/m²; normal weight, 18.5 kg/m² ≤ BMI < 24.0 kg/m²; overweight, 24.0 kg/m² ≤ BMI < 28.0 kg/m²; and obese, BMI ≥ 28.0 kg/m². The recommended total GWG ranges were 11.0–16.0 kg for underweight women, 8.0–14.0 kg for normal-weight women, 7.0–11.0 kg for overweight women, and 5.0–9.0 kg for obese women.

To minimize bias caused by differences in gestational age at delivery, especially among women with preterm birth, GWG was classified using a gestational-age-adjusted approach. The recommended first-trimester GWG range was 0–2.0 kg for all BMI categories. The BMI-specific recommended weekly GWG ranges during the second and third trimesters were 0.37–0.56, 0.26–0.48, 0.22–0.37, and 0.15–0.30 kg/week for underweight, normal-weight, overweight, and obese women, respectively. For each participant, the expected GWG range at delivery was calculated as the recommended first-trimester GWG range plus the BMI-specific weekly GWG range multiplied by the number of gestational weeks after 13 weeks. Participants were classified as having insufficient, adequate, or excessive GWG if their observed GWG was below, within, or above the corresponding gestational-age-specific recommended range, respectively. The recommended GWG range in the first trimester was 0–2.0 kg for all BMI categories.

### Data collection

Data were extracted from the hospital electronic medical record system by two trained investigators who had completed standardized training and assessment. The collected variables included maternal baseline characteristics, delivery and perinatal outcomes, neonatal outcomes, pregnancy-related complications, and postpartum complications. Maternal baseline characteristics included age, gravidity, parity, educational level, height, pre-pregnancy weight, pre-pregnancy BMI, and GWG. Delivery and perinatal outcomes included mode of delivery, premature rupture of membranes, and preterm birth. Mode of delivery was classified as vaginal delivery or cesarean delivery. Neonatal outcomes included birth weight, admission to the neonatal intensive care unit (NICU), and Apgar score.

### Statistical analysis

Statistical analyses were performed using SPSS version 27.0. Categorical variables were presented as numbers and percentages [*n* (%)], and between-group comparisons were performed using the chi-square test or Fisher’s exact test, as appropriate. Continuous variables were first tested for normality. Normally distributed variables were expressed as mean ± standard deviation (SD) and compared using one-way analysis of variance (ANOVA). Non-normally distributed variables were expressed as median and interquartile range (IQR) and compared using the Kruskal-Wallis H test.Variables that were statistically significant in the univariate analysis or considered clinically relevant were included in multivariable logistic regression models.

### Diagnostic criteria for pregnancy complications and adverse outcomes

1. Preeclampsia: Preeclampsia was diagnosed based on the criteria from the American College of Obstetricians and Gynecologists (ACOG) guidelines, defined as new-onset hypertension (blood pressure ≥ 140/90 mmHg) at or after 20 weeks of gestation accompanied by proteinuria, or in the absence of proteinuria, new-onset hypertension with new-onset end-organ dysfunction [14, 15].
2. Postpartum anemia: Postpartum anemia was defined as a hemoglobin (Hb) concentration of less than 100 g/L measured within 24 to 48 h after delivery [16].
3. Puerperal infection: Puerperal infection was defined as infection of the genital tract occurring after delivery and during the postpartum hospitalization, diagnosed on the basis of fever, uterine tenderness, foul-smelling lochia, leukocytosis, or clinician-documented infection requiring treatment.
4. PROM : In the main analysis, PROM was defined as rupture of membranes before the onset of labor, regardless of gestational age, including both term PROM and PPROM. PPROM was defined as rupture of membranes before labor and before 37 completed weeks of gestation [17]. Because of the limited number of PPROM cases, PROM and PPROM were not modeled separately in the primary regression analysis. A sensitivity analysis restricted to term pregnancies was performed to assess the potential influence of preterm delivery and PPROM.
5. Hypothyroidism: Hypothyroidism during pregnancy was defined according to pregnancy-specific and assay-specific reference ranges for thyroid function tests [18]. Overt hypothyroidism was defined as an elevated serum thyroid-stimulating hormone concentration accompanied by a decreased free thyroxine concentration, or a TSH concentration ≥ 10 mIU/L regardless of FT4 level. Subclinical hypothyroidism was defined as an elevated TSH concentration with a normal FT4 concentration. When pregnancy-specific reference ranges were unavailable, an upper TSH reference limit of approximately 4.0 mIU/L was used, in accordance with the 2017 American Thyroid Association guideline.
6. Preterm birth was defined as delivery before 37 completed weeks of gestation [19]. Because iatrogenic preterm births due to severe maternal complications were excluded, preterm birth in the present analysis mainly referred to spontaneous or non-iatrogenic preterm birth.

## Results

### Distribution of GWG among women with different pre-pregnancy BMI

According to the gestational-age-adjusted GWG classification based on WS/T 801–2022, 112 women (15.7%), 282 women (39.5%), and 320 women (44.8%) were classified as having insufficient, adequate, and excessive GWG, respectively.

The baseline characteristics of the participants according to gestational-age-adjusted GWG category are presented in Table 1. Maternal age, the proportion of women aged ≥ 40 years, gravidity, primiparity, previous cesarean delivery, use of assisted reproductive technology, educational level, and regular prenatal visits did not differ significantly among the three groups. However, pre-pregnancy BMI differed significantly across GWG categories (*P* < 0.001), with the highest mean BMI observed in the excessive GWG group and the lowest in the insufficient GWG group. The distribution of pre-pregnancy BMI categories also differed significantly among the groups (*P* < 0.001), with higher proportions of overweight and obese women in the excessive GWG group. Gestational age at delivery also differed significantly among the three groups, with the lowest mean gestational age observed in the insufficient GWG group.

**Table 1:** Baseline and pregnancy characteristics of participants according to gestational-age-adjusted gestational weight gain category

Characteristic	Total cohort *n* = 714	Insufficient GWG *n* = 112	Adequate GWG *n* = 282	Excessive GWG *n* = 320	*P* value
Maternal age, years	37.6 ± 2.4	37.7 ± 2.5	37.5 ± 2.3	37.6 ± 2.4	0.812
Age ≥ 40 years	126 (17.6)	22 (19.6)	47 (16.7)	57 (17.8)	0.768
Pre-pregnancy BMI, kg/m²	23.4 ± 3.1	21.8 ± 2.5	22.9 ± 2.8	24.3 ± 3.2	< 0.001
Pre-pregnancy BMI category					< 0.001
Underweight	43 (6.0)	17 (15.2)	19 (6.7)	7 (2.2)	
Normal weight	417 (58.4)	79 (70.5)	187 (66.3)	151 (47.2)	
Overweight	204 (28.6)	15 (13.4)	65 (23.0)	124 (38.8)	
Obese	50 (7.0)	1 (0.9)	11 (3.9)	38 (11.9)	
Gravidity	2.3 ± 1.1	2.2 ± 1.0	2.3 ± 1.1	2.4 ± 1.2	0.296
Primiparity	297 (41.6)	45 (40.2)	119 (42.2)	133 (41.6)	0.941
Previous cesarean delivery	244 (34.2)	37 (33.0)	93 (33.0)	114 (35.6)	0.792
Assisted reproductive technology	54 (7.6)	9 (8.0)	21 (7.4)	24 (7.5)	0.982
Educational level ≥ college	398 (55.7)	60 (53.6)	160 (56.7)	178 (55.6)	0.862
Gestational age at delivery, weeks	38.4 ± 1.3	38.1 ± 1.5	38.5 ± 1.2	38.4 ± 1.3	0.028

### Comparison of maternal outcomes among women with different gestational-age-adjusted gestational weight gain categories

Maternal outcomes according to gestational-age-adjusted GWG category are shown in Table 2. The incidence of premature rupture of membranes differed significantly among the three groups, being highest in the insufficient GWG group. Preterm birth was also more frequent among women with insufficient GWG. Cesarean delivery and postpartum anemia were more common in the excessive GWG group. No significant differences were observed in postpartum hemorrhage or postpartum infection among the three groups.

**Table 2:** Maternal outcomes according to gestational-age-adjusted GWG category

Maternal outcome	Total cohort *n* = 714	Insufficient GWG *n* = 112	Adequate GWG *n* = 282	Excessive GWG *n* = 320	*P* value
Premature rupture of membranes	148 (20.7)	31 (27.7)	48 (17.0)	69 (21.6)	0.032
Preterm birth	58 (8.1)	15 (13.4)	18 (6.4)	25 (7.8)	0.049
Cesarean delivery	421 (59.0)	61 (54.5)	154 (54.6)	206 (64.4)	0.026
Vaginal delivery	293 (41.0)	51 (45.5)	128 (45.4)	114 (35.6)	0.026
Postpartum hemorrhage	37 (5.2)	7 (6.3)	13 (4.6)	17 (5.3)	0.794
Postpartum anemia	119 (16.7)	19 (17.0)	35 (12.4)	65 (20.3)	0.035
Postpartum infection	26 (3.6)	5 ( 4.5)	9 (3.2)	12 (3.8)	0.816

### Neonatal outcomes according to gestational-age-adjusted gestational weight gain category

Neonatal outcomes are presented in Table 3. Neonatal birth weight differed significantly among the three GWG groups, with the highest mean birth weight observed in the excessive GWG group and the lowest in the insufficient GWG group. The incidences of low birth weight and small for gestational age were higher in the insufficient GWG group, whereas macrosomia and large for gestational age were more frequent in the excessive GWG group. No significant differences were found in 5-min Apgar score < 8 or NICU admission among the three groups.

**Table 3:** Neonatal outcomes according to gestational-age-adjusted GWG category

Neonatal outcome	Total cohort *n* = 714	Insufficient GWG *n* = 112	Adequate GWG *n* = 282	Excessive GWG *n* = 320	*P* value
Birth weight, g	3261 ± 462	3120 ± 438	3235 ± 421	3370 ± 474	< 0.001
Low birth weight	43 (6.0)	13 (11.6)	14 (5.0)	16 (5.0)	0.018
Macrosomia	54 (7.6)	4 (3.6)	16 (5.7)	38 (11.9)	0.004
Small for gestational age	52 (7.3)	14 (12.5)	20 (7.1)	18 (5.6)	0.041
Large for gestational age	73 (10.2)	6 (5.4)	24 (8.5)	43 (13.4)	0.026
5-min Apgar score < 8	12 (1.7)	3 (2.7)	4 (1.4)	5 (1.6)	0.669
NICU admission	62 (8.7)	12 (10.7)	22 (7.8)	28 (8.8)	0.651

### Pregnancy complications according to gestational-age-adjusted gestational weight gain category

Pregnancy complications according to GWG category are shown in Table 4. The incidence of preeclampsia differed significantly among the three groups and was highest in the excessive GWG group. Gestational hypothyroidism was more frequent in the insufficient GWG group. However, no significant differences were observed in hypertensive disorders of pregnancy, gestational diabetes mellitus, placenta previa, placental abruption, or intrahepatic cholestasis of pregnancy.

**Table 4:** Maternal complications and pregnancy-related conditions according to gestational weight gain category

Maternal pregnancy-related conditions	Total cohort *n* = 714	Insufficient GWG *n* = 112	Adequate GWG *n* = 282	Excessive GWG *n* = 320	*P* value
Hypertensive disorders of pregnancy	83 (11.6)	12 ( 10.7)	28 (9.9)	43 (13.4)	0.378
Gestational diabetes mellitus	112 (15.7)	15 (13.4)	39 (13.8)	58 (18.1)	0.225
Pregnancy complicated by hypothyroidism	92 (12.9)	22 (19.6)	34 (12.1)	36 (11.3)	0.041
Placenta previa	31 (4.3)	4 ( 3.6)	12 (4.3)	15 (4.7)	0.886
Placental abruption	9 (1.3)	2 (1.8)	3 (1.1)	4 (1.3)	0.834
Intrahepatic cholestasis of pregnancy	25 (3.5)	5 (4.5)	9 (3.2)	11 (3.4)	0.820

### Multivariable logistic regression analysis of gestational-age-adjusted gestational weight gain categories and adverse pregnancy outcomes

In the multivariable logistic regression analysis, adequate GWG was used as the reference category. After adjustment for potential confounders, insufficient GWG was associated with higher odds of premature rupture of membranes, hypothyroidism during, and low birth weight. Excessive GWG was associated with higher odds of postpartum anemia, preeclampsia and macrosomia. The association between insufficient GWG and preterm birth was attenuated after applying the gestational-age-adjusted GWG classification and was no longer statistically significant. The corresponding results are presented in Table 5.

**Table 5:** Multivariate logistic regression analysis of associations between gestational-age-adjusted GWG category and adverse pregnancy outcomes

Pregnancy-related outcomes	Insufficient GWGᵃ			Excessive GWGᵃ		
	Adjusted OR	95%CI	*P* value	Adjusted OR	95%CI	*P* value
PROM	1.65	1.01–2.69	0.046	1.23	0.81–1.86	0.334
Preterm birth	1.82	0.93–3.56	0.081	1.28	0.70–2.33	0.422
Postpartum anemia	1.31	0.72–2.39	0.377	1.78	1.12–2.84	0.015
Preeclampsia	1.04	0.46–2.37	0.925	1.71	1.01–2.89	0.046
Gestational hypothyroidism	1.89	1.08–3.30	0.026	0.94	0.56–1.57	0.808
Low birth weight	2.12	1.05–4.29	0.036	1.01	0.50–2.05	0.978
Macrosomia	0.64	0.20–2.05	0.455	2.12	1.16–3.88	0.015

*Abbreviations*: The reference group was “adequate GWG”

Adequate GWG was the reference category. Models were adjusted for maternal age, pre-pregnancy BMI category, parity, previous cesarean delivery, mode of conception, educational level, hypertensive disorders of pregnancy, and number of prenatal visits; neonatal sex was additionally adjusted for in analyses of neonatal outcomes

### Sensitivity analysis

In the sensitivity analysis restricted to term pregnancies, 656 women remained after excluding those who delivered before 37 completed weeks of gestation. The corresponding results are presented in Table 6. The overall pattern of associations was generally consistent with that observed in the main analysis. Excessive GWG remained associated with higher odds of cesarean delivery (aOR: 1.45, 95% CI: 1.01–2.10, *P* = 0.044), postpartum anemia (aOR: 1.76, 95% CI: 1.08–2.86, *P* = 0.023), and macrosomia (aOR: 2.05, 95% CI: 1.10–3.83, *P* = 0.024). Insufficient GWG remained associated with higher odds of hypothyroidism during pregnancy (aOR: 1.82, 95% CI: 1.01–3.29, *P* = 0.047). In contrast, the associations of insufficient GWG with premature rupture of membranes and low birth weight were attenuated and were no longer statistically significant. The association between excessive GWG and preeclampsia was also attenuated after restriction to term pregnancies.

**Table 6:** Sensitivity analysis restricted to term pregnancies

Pregnancy-related outcomes	Insufficient GWGᵃ			Excessive GWGᵃ		
	Adjusted OR	95%CI	*P* value	Adjusted OR	95%CI	*P* value
PROM	1.52	0.90–2.58	0.119	1.19	0.77–1.85	0.431
Cesarean delivery	0.98	0.61–1.57	0.931	1.45	1.01–2.10	0.044
Postpartum anemia	1.24	0.66–2.34	0.506	1.76	1.08–2.86	0.023
Preeclampsia	0.96	0.39–2.33	0.926	1.69	0.98–2.93	0.059
Hypothyroidism during pregnancy	1.82	1.01–3.29	0.047	0.91	0.53–1.56	0.733
Low birth weight	1.76	0.74–4.20	0.203	0.98	0.42–2.27	0.960
Macrosomia	0.66	0.20–2.17	0.493	2.05	1.10–3.83	0.024

*Abbreviations*: The reference group was “adequate GWG”

Adequate GWG was the reference category. Models were adjusted for maternal age, pre-pregnancy BMI category, parity, previous cesarean delivery, mode of conception, educational level, hypertensive disorders of pregnancy, and number of prenatal visits; neonatal sex was additionally adjusted for in analyses of neonatal outcomes

## Discussion

With the progressive delay in childbearing age, pregnancies among women of advanced maternal age have become an increasingly important public health concern in obstetric practice. In this context, gestational weight gain, as a modifiable factor during pregnancy, may play an important role in maternal and neonatal health. The present study showed that deviations from the recommended GWG range were common among AMA women and were associated with several adverse pregnancy outcomes. After multivariable adjustment, inadequate GWG was independently associated with increased risks of premature rupture of membranes, hypothyroidism complicating pregnancy, and low birth weight, whereas excessive GWG was independently associated with increased risks of postpartum anemia, preeclampsia, and macrosomia. These findings suggest that both insufficient and excessive GWG may contribute to adverse pregnancy outcomes in AMA pregnancies, highlighting the clinical importance of appropriate weight management during pregnancy [20].

Notably, excessive GWG appeared to have a significant impact on maternal metabolic and hypertensive complications, particularly preeclampsia. This finding may be partly explained by reduced metabolic adaptability in women of advanced maternal age, together with excessive fat accumulation during pregnancy [21–23]. Previous studies have suggested that excessive GWG can exacerbate insulin resistance and promote systemic inflammatory responses, which may impair endothelial function and increase the risk of hypertensive disorders of pregnancy [24]. In addition, excessive weight gain during pregnancy may increase maternal blood volume and circulatory load, placing greater stress on the maternal cardiovascular system and potentially contributing to pregnancy-related complications [25]. In women of AMA, declines in basal metabolic capacity and physiological regulatory function may make these metabolic and hemodynamic alterations more pronounced, thereby further amplifying the adverse effects associated with excessive GWG [26].

In the present study, excessive GWG was also independently associated with postpartum anemia. Although the underlying mechanism remains uncertain, this association may be partly related to the higher rate of cesarean delivery observed among women with excessive GWG, as operative delivery may increase blood loss and contribute to postpartum anemia. Excessive GWG may also reflect metabolic dysregulation, suboptimal dietary quality, or dilutional changes during pregnancy, all of which may influence maternal hematologic status. However, postpartum anemia is multifactorial and may also be affected by antepartum anemia, iron supplementation, delivery mode, and intrapartum blood loss. Therefore, this finding should be interpreted with caution and confirmed in future studies with more detailed hematologic and nutritional data.

In addition to excessive weight gain, the present study found that inadequate GWG was significantly associated with adverse outcomes, particularly premature rupture of membranes, hypothyroidism complicating pregnancy, and low birth weight. Unlike excessive GWG, which mainly reflects increased metabolic burden, inadequate GWG may indicate insufficient nutritional intake or limited maternal energy reserves during pregnancy [27, 28]. Among women of advanced maternal age, age-related declines in nutrient absorption efficiency and metabolic regulation may make them more susceptible to inadequate energy supply during gestation. Such conditions may impair placental development and compromise the structural integrity of the fetal membranes, thereby increasing the risk of premature rupture of membranes [29]. Furthermore, insufficient intake of energy, protein, iodine, selenium, iron, and other micronutrients may affect thyroid hormone synthesis and metabolism, potentially contributing to thyroid dysfunction during pregnancy [30]. Insufficient GWG was associated with hypothyroidism during pregnancy; however, this finding should be interpreted cautiously. Because the timing of thyroid dysfunction diagnosis could not be fully determined in this retrospective study, the temporal direction of this association remains unclear. Thyroid dysfunction itself may affect maternal metabolism and weight change, and reverse causation cannot be excluded.Therefore, in AMA pregnancies, inadequate GWG may not only reflect suboptimal nutritional status but also serve as a potential indicator of underlying metabolic or endocrine disturbance.

The neonatal findings further support the importance of maintaining GWG within an appropriate range. Inadequate GWG was associated with lower birth weight and independently increased the risk of low birth weight, whereas excessive GWG was associated with higher birth weight and independently increased the risk of macrosomia [31]. These results are consistent with the biological role of maternal nutritional supply in fetal growth. Insufficient maternal weight gain may limit fetal substrate availability and increase the risk of fetal growth restriction, while excessive GWG may promote fetal overgrowth through increased glucose, lipid, and inflammatory exposure. Although the rates of low Apgar score and NICU admission did not differ significantly among GWG groups, abnormal fetal growth itself may have important implications for both short-term neonatal outcomes and long-term offspring health.

Because total GWG is inherently affected by gestational age at delivery, women who deliver preterm have less time to gain weight, which may introduce reverse causality or classification bias when preterm birth is analyzed as an outcome. In the present study, preterm birth was more frequent in the inadequate GWG group in the unadjusted analysis; however, this association did not remain statistically significant after multivariable adjustment. To further address the potential influence of pregnancy duration, we performed a sensitivity analysis restricted to term pregnancies. The results were generally consistent with the primary analyses, suggesting that the observed associations between GWG categories and most maternal and neonatal outcomes were relatively robust. Nevertheless, the interpretation of preterm birth should remain cautious, and future studies using gestational-age-standardized GWG indicators or GWG rate are warranted.

GWG may also have implications beyond immediate perinatal outcomes. In the present study, inadequate GWG was independently associated with low birth weight, whereas excessive GWG was independently associated with macrosomia, suggesting that maternal weight gain during pregnancy may influence fetal growth in different directions. Previous studies have shown that abnormal maternal weight status and excessive GWG may affect offspring metabolic programming, partly through alterations in the intrauterine nutritional and inflammatory environment. Emerging evidence also suggests that excessive GWG may influence the neonatal metabolic milieu and infant gut microbiota, including changes in beneficial bacteria, opportunistic pathogens, and metabolic pathways, which may be related to accelerated weight gain in early childhood [31–33]. Although long-term offspring outcomes were not assessed in the present study, these findings support the importance of maintaining GWG within the recommended range to optimize both short-term neonatal growth and potential long-term child health.

From a clinical perspective, the findings of this study suggest that greater attention should be paid to dynamic monitoring of maternal weight during pregnancy in women of AMA [34, 35]. We believe that the general BMI-based framework for GWG management used in women younger than 35 years remains applicable to AMA women. However, because AMA women have higher risks of metabolic disorders, hypertensive complications, thyroid dysfunction, placental insufficiency, and abnormal fetal growth, the same routine management strategy may be insufficient if applied without individualized risk assessment. Therefore, GWG management in AMA pregnancies should begin before conception or in early pregnancy and should include assessment of prepregnancy BMI, blood pressure, glucose metabolism, thyroid function, anemia status, diet, and physical activity. GWG should be monitored dynamically throughout pregnancy. For excessive GWG, dietary counseling, appropriate physical activity, and closer monitoring of blood pressure and metabolic indicators are recommended. For inadequate GWG, nutritional assessment, individualized dietary guidance, fetal growth monitoring, and evaluation of placental function should be strengthened. Thus, AMA women may not require completely separate GWG cutoffs solely based on age, but they do require a more intensive and individualized management strategy within the existing guideline framework.

## Limitations

Several limitations of this study should be acknowledged. First, GWG was assessed using total gestational weight gain throughout pregnancy, which may be influenced by differences in gestational duration. Although sensitivity analyses restricted to term pregnancies were performed, future studies using gestational-age-standardized GWG or GWG rate are warranted to further validate these findings. Second, because some medically indicated preterm births resulting from severe maternal complications were excluded, the findings related to preterm birth should be interpreted with caution and should not be generalized to all types of preterm birth. Finally, owing to the retrospective nature of the data, this study was unable to fully control for potential confounding factors such as assisted reproductive technology, dietary intake, physical activity, and socioeconomic factors. Larger multicenter prospective studies are needed to further validate these findings.

## Conclusions

In conclusion, both inadequate and excessive GWG were associated with adverse pregnancy outcomes among women of advanced maternal age. Inadequate GWG was mainly associated with premature rupture of membranes, hypothyroidism complicating pregnancy, and low birth weight, whereas excessive GWG was mainly associated with postpartum anemia, preeclampsia, and macrosomia. These findings emphasize the importance of individualized GWG monitoring and intervention in AMA pregnancies to improve maternal and neonatal outcomes.

## Data Availability

The datasets generated and analysed during the current study are not publicly available due to the privacy of patients but are available from the corresponding author on reasonable request.

## Abbreviations

AMA: Advanced maternal age
GWG: Gestational weight gain
BMI: Body mass index
PROM: Premature rupture of membranes
PPROM: Preterm prelabor rupture of membranes
NICU: Neonatal intensive care unit
ACOG: American College of Obstetricians and Gynecologists guidelines
Hb: Hemoglobin
SD: Standard deviation
OR: Odds ratio
aOR: Adjusted odds ratio
CI: Confidence interval
ANOVA: Analysis of variance
